# Intracystic Papillary Carcinoma in the Male Breast: A Rare Endpoint of a Wide Spectrum

**DOI:** 10.1155/2013/129353

**Published:** 2013-08-26

**Authors:** Ketan Vagholkar, Khojasteh Dastoor, Indumati Gopinathan

**Affiliations:** ^1^Department of Surgery, Dr. D. Y. Patil Medical College, Navi Mumbai 400706, Maharashtra state, India; ^2^Annapurna Niwas, 229 Ghantali Road, Thane 400602, Maharashtra State, India; ^3^Clinicopathological Laboratory, Institute of Pathology, Mumbai 400089, Maharashtra State, India

## Abstract

*Introduction*. Fibrocystic disease of the male breast is uncommon. The presence of a spectrum of changes ranging from fibrocystic disease to duct papilloma to papillary carcinoma in the same patient renders the case a rarity and therefore reportable. *Case Report*. A case of intracystic papillary carcinoma of the male breast is presented. *Discussion*. The pathological, clinical, diagnostic, and therapeutic options are discussed after reviewing the literature. *Conclusion*. Modified radical mastectomy with axillary clearance is the safest option for established cases.

## 1. Introduction

Intracystic papillary carcinoma of the male breast is an extremely rare entity accounting for less than 1% of all breast malignancies [1]. The condition is extremely rare even in males with very few case reports in English literature. This rare condition in the majority of cases is noninvasive, leading to both diagnostic and therapeutic dilemmas. A case of intracystic papillary carcinoma in a 55-year-old male is presented in view of the presence of the entire spectrum ranging from fibrocystic disease to intraductal papilloma to intracystic papillary carcinoma on histology of the specimen along with review of the literature. 

## 2. Case Report

**Figure 1:**
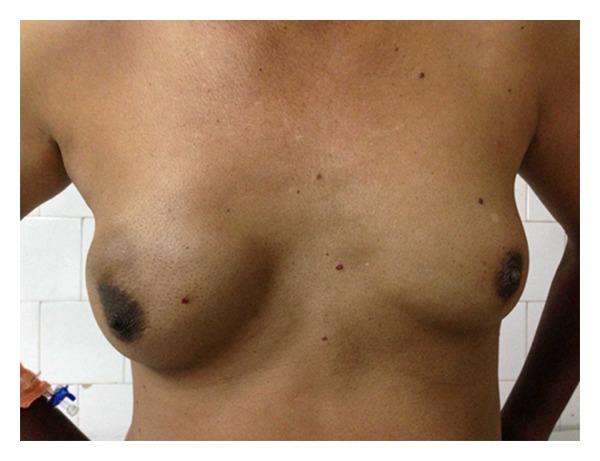
Clinical photograph of the right breast showing the mass.

**Figure 2:**
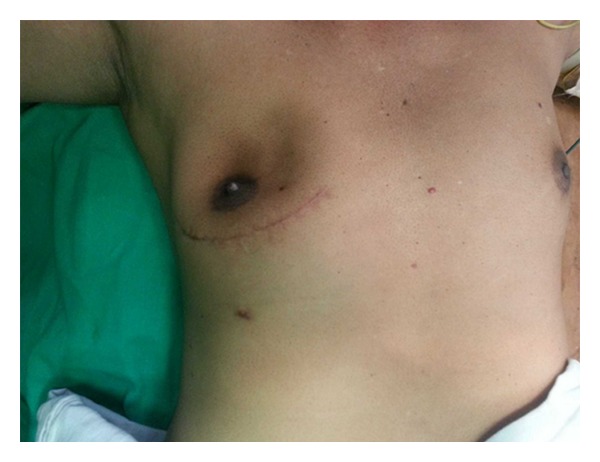
Excision biopsy of the lump done through an elliptical inframammary incision.

**Figure 3:**
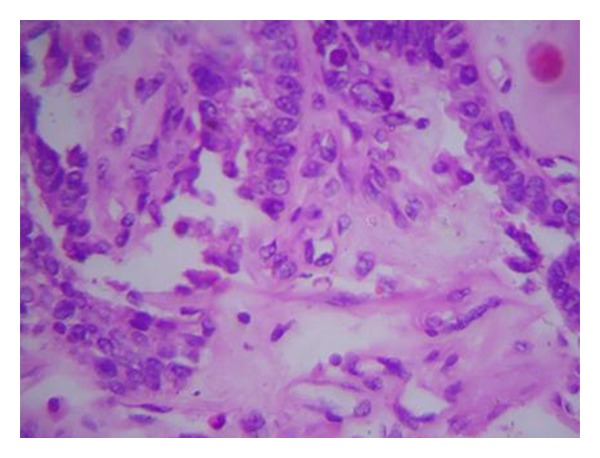
Histopathology of the excision biopsy specimen shows infiltrating papillary carcinoma with atypical nuclei, chromatin clumping, and severe pleomorphism (H&E staining, magnification 40x).

**Figure 4:**
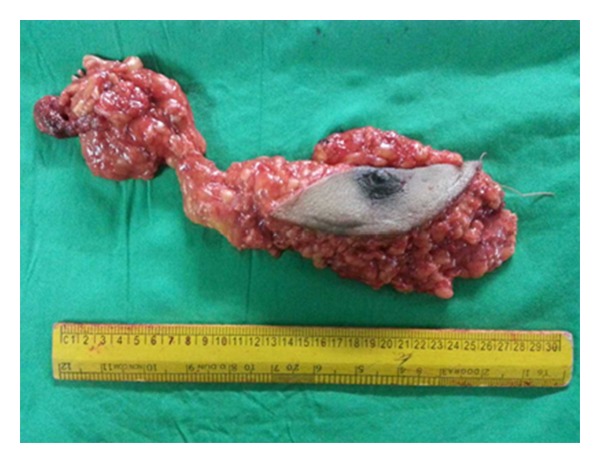
Specimen of the completion of modified radical mastectomy with ipsilateral axillary clearance.

**Figure 5:**
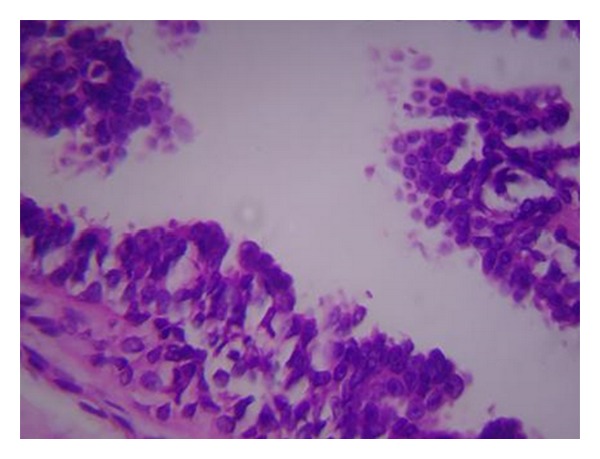
Duct papilloma (H&E staining, magnification 40x).

A 55-year-old male presented with history of a right sided breast mass since 1 year; however over the past 6 months the mass has rapidly increased in size. Patient also gave history of intermittent episodes of blood stained nipple discharge. There was no history of a similar lesion in the opposite breast nor was there any lesion in the ipsilateral axilla. Physical examination revealed a bosselated mass underlying the right nipple areolar complex measuring 7 cm in diameter with a bosselated appearance and variegated consistency (Figure 1). The mass was free from the underlying muscle. An FNAC was done which revealed extensive fibrocystic disease with suspicious papillary hyperplasia or malignancy. Cytological examination of the nipple discharge did not reveal any malignant cells. In view of the equivocal reports an open excision biopsy was done through an inframammary elliptical incision (Figure 2). The whole mass was excised. Histological evaluation of the specimen revealed a cystic mass typical of fibrocystic disease with papillary excrescences within the cysts. A papillary carcinoma with invasive component was picked up in one of the cysts (Figure 3). The resection margins were found to be free of tumour. The tumour was oestrogen receptor positive. In view of the invasive component detected on histology the patient underwent a completion modified radical mastectomy with ipsilateral axillary clearance (Figure 4). The specimen of completion mastectomy revealed an intraductal papilloma with negative axillary lymph nodes and no evidence of any residual tumour (Figure 5). Postoperative recovery was uneventful. The patient has been followed up for a period of 6 months with no evidence of any local or regional recurrence. 

## 3. Discussion

Male breast cancer is by itself a rare disease. Papillary lesions of the male breast are an extreme rarity. These comprise a spectrum of lesions ranging from benign intraductal papillomas to intraductal papillary carcinomas and invasive papillary carcinoma [2]. The literature on this topic is rife as not many such cases have been found. Majority of cases are in the form of isolated case reports. As a result there is no clear consensus on the diagnosis and treatment of this disease. 

The commonest presentation is growing fullness in the breast or gynaecomastia in males. Nipple discharge can also be a presenting symptom [2]. 

FNAC in cystic lesions of the breast may not offer the same sensitivity and specificity as it does in solid lesions of the breast [3]. This could lead to a misdiagnosis of a cancer. Hence it would be a safe practice to perform an excision biopsy which will allow to elaborate histological evaluation including immunohistochemical studies [3, 4]. 

Intracystic papillary carcinoma was once regarded as a purely intraductal neoplasm. However recent evidence suggests that it could be invasive as it lacks myoepithelial lining. Studies on immunohistochemical analysis of these tumours have revealed that all these tumours are oestrogen receptor positive, HER2 negative whereas most are progesterone receptor positive. This confers good prognosis to this tumour [4]. 

Spread to axillary lymph nodes has not been reported in any of the cases reported in the literature; therefore the need for either a sentinel node biopsy or an axillary clearance remains debatable. However in view of the invasive potential it would be a safe practice to either do a sentinel biopsy or an axillary clearance [5, 6]. In the case presented, as histology revealed features of invasiveness, a completion modified radical mastectomy with axillary clearance was done. As the axilla was found to be negative, the need for adjuvant therapy did not arise. The detection of an intraductal papilloma in the case presented highlights the entire spectrum of changes in a fibrocystic disease of the breast ranging from cyst formation, intraductal papillomatosis, and papillary carcinoma finally leading to invasive papillary carcinoma [7, 8]. The debate still continues as to the choice of surgical procedure. Older case reports suggested simple mastectomy as the procedure of choice [8]. However in view of its invasive potential as has been studied recently a modified radical mastectomy with ipsilateral axillary clearance would be the ideal surgical procedure [8]. This would retain the principle of radicality for an invasive cancer thereby reducing the chances of local recurrence and metastasis to the verge of extinction. The prognosis remains excellent in such cases [9, 10]. 

## 4. Conclusion 

Intracystic papillary carcinoma is an extremely rare lesion of the male breast. A diagnostic excision biopsy followed by a modified radical mastectomy with axillary clearance is the mainstay of treatment.
